# Impact of Anemia on Cardiovascular Events and All-Cause Death Among Participants Who Received Intense Blood Pressure Treatment: A Secondary Analysis of SPRINT

**DOI:** 10.31083/j.rcm2501006

**Published:** 2024-01-08

**Authors:** Xiaochuan Liu, Beiru Lin, Sichen Yao, Zhigang Pan

**Affiliations:** ^1^Department of General Practice, Zhongshan Hospital, Fudan University, 200032 Shanghai, China; ^2^Department of General Practice, Hainan West Central Hospital, 571700 Danzhou, Hainan, China; ^3^Department of General Practice, Wujing Community Health Service Center, 200241 Shanghai, China; ^4^Center of Community-Based Health Research, Fudan University, 200032 Shanghai, China

**Keywords:** SPRINT, blood pressure, intensive, anemia, low hemoglobin, cardiovascular disease

## Abstract

**Background:**

To investigate whether anemia is associated with incident cardiovascular events and all-cause death among participants who received intensive blood pressure (BP) treatment in the Systolic Blood Pressure Intervention Trial (SPRINT).

**Methods:**

A total of 4394 participants who received intensive BP control (systolic BP <120 mmHg) in SPRINT were included. Anemia status was self-reported. Our primary outcome was a composite of cardiovascular events, and the secondary outcome was all-cause death. Cox regression was used to compare the incidence of outcomes between participants with anemia and non-anemia. In order to balance the baseline characteristics between the 2 groups, inverse probability of treatment weighting (IPTW) was applied. Hazard ratios (HRs), along with 95% confidence intervals (CIs), were then calculated.

**Results:**

There were 4394 participants who received intensive BP control (537 participants with anemia). Participants with anemia were older (mean age 68.86 versus 67.75, *p *= 0.01) and more likely to be female (64.8% versus 31.8%, *p* < 0.001). The presence of anemia was strongly associated with composite cardiovascular events after adjusting for potential confounders (HR 1.66, 95% CI 1.18–2.34, *p* = 0.004). The association remained statistically significant even in the population after IPTW (HR 1.55, 95% CI 1.06–2.27, *p* = 0.024). The secondary outcome revealed that participants with anemia had a higher rate of all-cause death compared to those without anemia. The HR of all-cause death for participants with anemia was 1.61 (95% CI 1.00–2.57, *p* = 0.049) in the population after IPTW.

**Conclusions:**

Anemia appears to be an independent risk factor for composite cardiovascular events and all-cause death among participants who received intensive BP control in SPRINT.

**Clinical Trial Registration:**

URL: https://www.clinicaltrials.gov; Unique identifier: NCT01206062. All SPRINT anonymized data can be found at the National Heart, Lung and Blood Institute (NHLBI) Biologic Specimen and Data Repository (https://biolincc.nhlbi.nih.gov/home/).

## 1. Introduction

Hypertension is a common chronic disease across the world [1]. Well-controlled blood pressure (BP) is associated with favorable cardiovascular health. Recently, a lower systolic blood pressure (SBP) control target has shown a beneficial effect in reducing cardiovascular events [2, 3, 4]. Among those trials related to intensive BP control, the Systolic Blood Pressure Intervention Trial (SPRINT) was designed with the lowest intensive SBP target (<120 mmHg). The main findings of SPRINT indicated intensive BP control reduced composite cardiovascular events and all-cause death by 25% and 27% compared to standard BP control (<140 mmHg). While intensive BP control has been shown to be associated with beneficial effects, it has also been linked to adverse events such as acute kidney injury, hypotension, syncope, and falls [2]. The hazard ratio (HR) for adverse events was higher in subgroups with advanced age or frailty [5]. Anemia, characterized by declined blood capacity of carrying oxygen, is not only a risk factor for cardiovascular diseases (CVD) [6, 7, 8] but also a prognosis predictor for adverse outcomes following heart failure and end-stage renal disease [9, 10, 11]. The prevalence of anemia increases with advancing age and exceeds 20% in the elderly population [12]. As the control target of SBP becomes lower, reduced blood supply with lower oxygen may be harmful to remote organs. In other words, anemia may be a risk factor for CVD among those who received intensive BP treatment. Although prior studies have found a positive link between anemia and compromised cerebral autoregulation and intensified myocardial ischemia [11, 13, 14], the impact of anemia on the risk of CVD among participants treated with lower SBP target has not yet been investigated. The low SBP control target of SPRINT gives us an opportunity to explore the relationship between anemia and the risk of CVD among participants enrolled in an intensive BP treatment group. We hypothesize that anemia could be a risk factor for CVD and all-cause death among participants who received intensive BP treatment in SPRINT.

## 2. Materials and Methods

### 2.1 Data Reproducibility Statement

All SPRINT anonymized data can be found at the National Heart, Lung and Blood Institute (NHLBI) Biologic Specimen and Data Repository (https://biolincc.nhlbi.nih.gov/home/).

### 2.2 Study Design and Population

This study was a secondary analysis of the SPRINT trial. As mentioned above, SPRINT was designed to investigate the beneficial effect of intensive BP control in reducing cardiovascular events, as compared with standard BP control. Details of the trial have been discussed elsewhere [15]. Briefly speaking, the SPRINT trial enrolled 9361 participants between November 2010 and March 2013. The participants were aged over 50 years and had an average SBP of 130 to 180 mmHg, along with an increased risk for CVD. This was defined as having at least one of the following: clinical or subclinical CVD other than stroke, a 10-year Framingham risk score (FRS) of 15% or higher, being aged 75 years or older, or having an estimated glomerular filtration rate (eGFR) of 20 to <60 mL/min/1.73 $m^{2}$.

Major exclusion criteria for the trial included diagnosed diabetes, prior stroke, severe chronic kidney disease (CKD) (eGFR <20 mL/min/1.73 $m^{2}$), congestive heart failure (ejection fraction <35%), or dementia. For the purpose of this study, only participants who received intensive blood pressure treatment were included.

This study was approved by the Institutional Review Board of each clinical site, and all participants provided informed consent.

### 2.3 Study Measurements

Baseline demographic data, including age, gender, and race were self-reported at randomization. Medical histories were recorded in a document called self-administered baseline history. As for anemia status, participants were asked if they had ever been told by a physician that they had anemia or low blood count. If they answered yes, then they were recorded as positive for anemia. The same kinds of questions were asked to collect other medical histories, including cancer, smoking status, and medication usage. In the SPRINT trial, prior CVD was defined as a history of clinical or subclinical CVD, including acute coronary syndrome, carotid revascularization, coronary revascularization, more than 50% stenosis of carotid/coronary/lower extremity artery, or a coronary artery calcium score of 400 or higher. Chronic kidney disease was defined as an eGFR of less than 60 mL/min/1.73 $m^{2}$. The eGFR was calculated using the modification of diet in renal disease 4-component equation [16]. BP was measured using an automated device (Omron-HEM-907, OMRON HEALTHCARE Co., Ltd., Kyoto, Japan) following standard procedures. The mean of 3 automated cuff readings was used for analysis. Laboratory specimens were obtained at baseline and stored at the University of Minnesota for estimation of serum markers, including blood glucose, serum creatinine, and lipid profiles.

### 2.4 Outcome Definitions

The primary outcome of our study was a composite of cardiovascular events, including nonfatal myocardial infarction, acute coronary syndrome not resulting in myocardial infarction, stroke, acute decompensated heart failure, or death from CVD. The definition of our primary outcome is consistent with the one used in SPRINT. Our secondary outcome was all-cause death, which is also a prespecified secondary outcome in SPRINT. All outcome events were reviewed and confirmed by experienced physicians who were blinded to the treatment assignment.

### 2.5 Statistical Analysis

All statistical analyses were performed using R version 3.6.2 (https://www.r-project.org/; R Foundation for Statistical Computing, Vienna, Austria). A *p* value less than 0.05 was considered to be statistically significant. Participants in the intensive BP treatment group were divided into 2 groups based on their anemia status. The baseline characteristics of the 2 groups were compared using appropriate statistical tests. Continuous variables were compared using the Wilcoxon rank sum test or 2-sample *t*-test, while categorical variables were compared using Pearson’s Chi-squared test or Fisher’s exact test. Cox proportional hazards models were used to compare the incidence of composite cardiovascular events and all-cause death between the 2 groups. HRs with 95% confidence intervals (CIs) were calculated, with participants without anemia serving as the reference group.

Since this was a secondary analysis of SPRINT, baseline characteristics may not be balanced between the 2 groups. One approach to help us remove confounding is the inverse probability of treatment weighting (IPTW) [17]. This method relies on building a logistic regression model to estimate the probability score of the exposure (anemia/non-anemia) observed for the study population and using the probability score as a weight in subsequent analyses. The predicted probability score was calculated by putting all our baseline characteristics into the logistic regression model. By applying these weights to the study population, we can create a pseudo-population in which baseline characteristics are balanced across the 2 groups. We constructed 4 Cox regression models to test the robustness of the relationship between anemia and outcomes. In model 1, no confounder was adjusted. In model 2, confounders with *p* value < 0.05 between the 2 groups were adjusted (age, gender, eGFR, SBP, diastolic BP, FRS, baseline CKD, race, serum creatinine, cancer history, cholesterol, blood glucose, and high-density lipoprotein). In model 3, we adjusted for the predicted probability score calculated by a logistic regression model. In model 4, the HR was calculated within the population after IPTW. A cumulative incidence plot for our outcomes was drawn both in pre-weighted and post-weighted populations with a log-rank test indicating the differences between the 2 groups. The follow-up time was censored at the end of the trial, death, loss to follow-up, or the occurrence of the outcomes being studied. The proportional hazards assumption was verified by checking the plot of Schoenfeld residuals.

Subgroup analyses were conducted to test the interaction effect (anemia status* subgroup) for our primary outcome among the following groups: age (≥75/<75 years), gender (male/female), FRS (≥15%/<15%), race (black/non-black), number of antihypertensive drugs at baseline (≥2/<2), previous CVD (Yes/No), medical history of cancer (Yes/No), SBP tertile at randomization (<132 mmHg; 132 to 145 mmHg; >145 mmHg), and baseline CKD (eGFR ≥60/<60 mL/min/1.73 $m^{2}$). As for the R packages we used in our study, tableone, survey, and reshape2 were used for the inverse probability of treatment weighting; survival and survminer were used for Cox proportional hazards models; forestplot was used to draw forest-plot.

### 2.6 Sensitivity Analysis

First, our hypothesis in this study is that anemia could be a risk factor for composite cardiovascular events and all-cause death among participants who received intensive BP treatment in SPRINT. To reinforce our findings, we also conducted the same analysis mentioned above among participants who received standard BP treatment. From our point of view, the relationship between anemia and outcomes found in the intensive BP treatment group may not exist in the standard BP treatment group. Second, we separated the whole SPRINT participants into 2 groups (anemia/non-anemia). The beneficial effect of intensive BP treatment compared with standard BP treatment in reducing cardiovascular events may not be identical among anemia and non-anemia participants. In other words, intensive BP treatment may not be suitable for participants with anemia.

## 3. Results

### 3.1 Characteristics of Study Participants

**Fig. 1.**
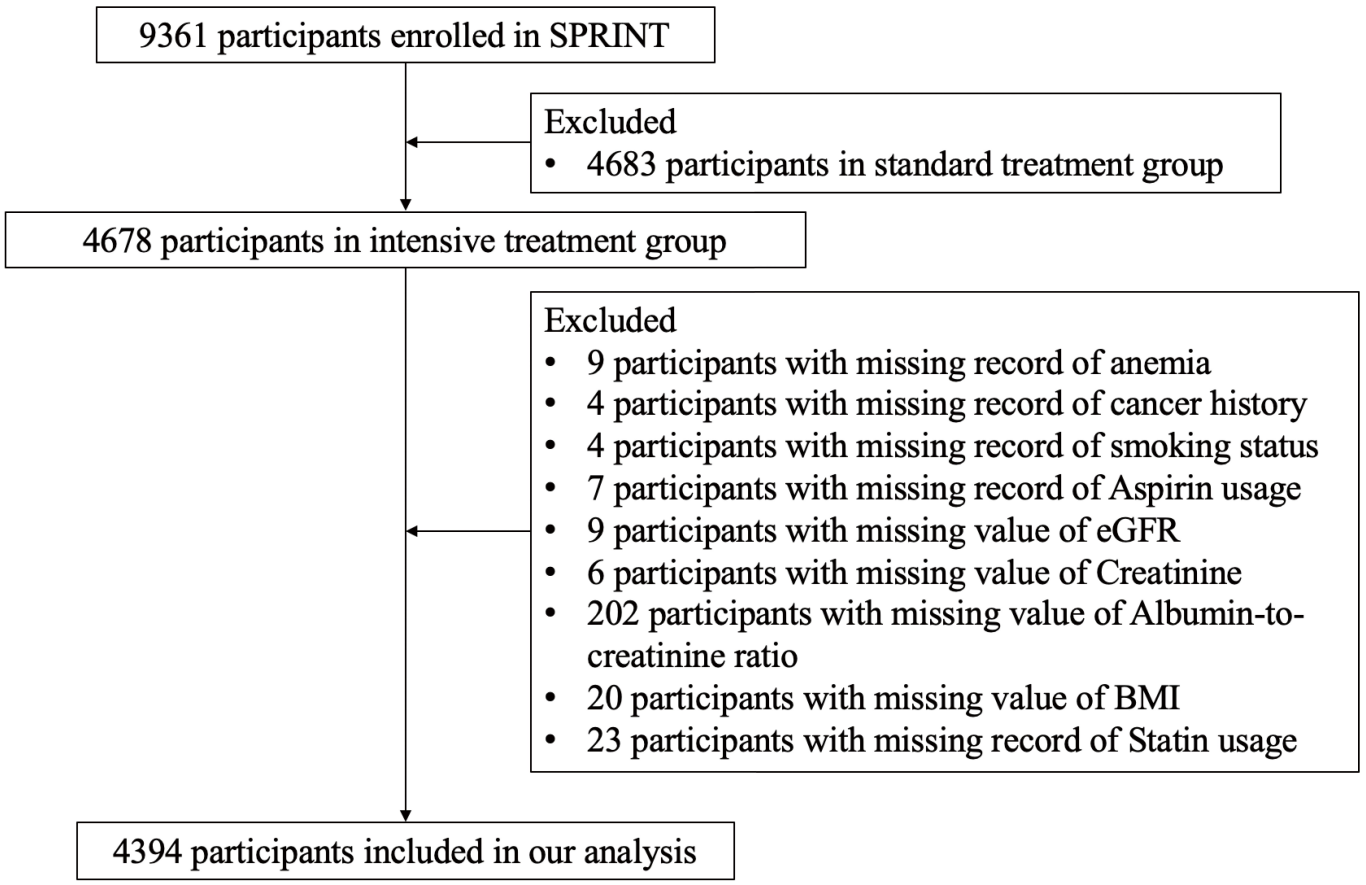
**Study flowchart**. SPRINT, Systolic Blood Pressure Intervention Trial; BMI, body mass index; eGFR, estimated glomerular filtration rates.

The study flowchart (Fig. 1) shows the enrollment process of our study. There were 9361 participants enrolled in SPRINT, with 4678 participants randomized to the intensive BP treatment group and 4683 participants randomized to the standard BP treatment group. For the purpose of this study, we excluded participants in the standard BP treatment group. We further excluded 9 participants with missing information about anemia, 4 participants with missing information about cancer history, 4 participants with missing information about smoking status, 7 participants with missing information about aspirin usage, 9 participants with missing value of eGFR, 6 participants with missing value about serum creatinine, 202 participants with missing value about urine albumin-to-creatinine ratio, 20 participants with missing value of body mass index (BMI), and 23 participants with missing information about statin usage. Finally, there were 4394 participants included in this study. The dynamic change of BP after randomization among participants with anemia and non-anemia was shown in **Supplementary Fig. 1**.

Table 1 shows the baseline characteristics between the 2 groups before and after IPTW. Participants with anemia were older (mean age 68.86 versus 67.75, *p* = 0.01), more likely to be female (64.8% versus 31.8%, *p* < 0.001), mostly Black race (37.2% versus 30.4%, *p* = 0.002), with lower FRS ≥15% (49.0% versus 63.4%, *p* < 0.001), a higher percentage of cancer history (17.1% versus 12.1%, *p* = 0.001), higher prevalence of baseline CKD (41.5% versus 27.1%, *p* < 0.001), higher SBP level at baseline (mean 140.96 mmHg versus 139.49, *p* = 0.043), lower DBP level at baseline (mean 76.96 versus 78.41, *p* = 0.008), lower eGFR (mean 66.15 versus 72.43, *p* < 0.001), higher serum creatinine (mean 1.12 versus 1.07, *p* = 0.001), higher cholesterol (mean 193.75 versus 189.92, *p* = 0.046), lower blood glucose (mean 97.01 versus 99.18, *p* = 0.001), and more elevated high-density lipoprotein (mean 55.78 versus 52.46, *p* < 0.001). After IPTW, baseline characteristics were similar between the 2 groups (Table 1, **Supplementary Fig. 2**).

**Table 1.** **Univariate comparison of baseline characteristics stratified by exposure to anemia in unweighted and propensity score-weighted participants**.

Characteristics		Unmatched			IPTW		
		Non-Anemia	Anemia	*p*	Non-Anemia	Anemia	*p*
N		3857	537		4393	4402.4	
Age		67.75 (9.32)	68.86 (9.82)	0.01	67.90 (9.36)	68.73 (9.39)	0.107
	<75	2794 (72.4)	360 (67.0)	0.011	3151.6 (71.7)	3030.4 (68.8)	0.25
	≥75	1063 (27.6)	177 (33.0)		1241.4 (28.3)	1372.1 (31.2)	
Gender (Female)		1226 (31.8)	348 (64.8)	<0.001	1573.5 (35.8)	1575.9 (35.8)	0.992
BMI, (${\mathrm{kg/m}}^{2}$)		29.95 (5.77)	29.78 (6.24)	0.538	29.92 (5.82)	29.44 (5.67)	0.099
SBP, mmHg		139.49 (15.72)	140.96 (15.86)	0.043	139.67 (15.84)	139.66 (14.91)	0.99
DBP, mmHg		78.41 (11.84)	76.96 (12.25)	0.008	78.23 (11.90)	77.48 (12.02)	0.28
SBP Tertile							
	<132 mmHg	1318 (34.2)	171 (31.8)	0.485	1486.6 (33.8)	1479.9 (33.6)	0.934
	132−145 mmHg	1232 (31.9)	172 (32.0)		1403.2 (31.9)	1374.0 (31.2)	
	>145 mmHg	1307 (33.9)	194 (36.1)		1503.2 (34.2)	1548.5 (35.2)	
FRS (≥15%)		2446 (63.4)	263 (49.0)	<0.01	2712.2 (61.7)	2813.0 (63.9)	0.409
Race (Black)		1174 (30.4)	200 (37.2)	0.002	1374.7 (31.3)	1351.1 (30.7)	0.812
Number of antihypertensive agents							
	<2	1474 (38.2)	212 (39.5)	0.606	1683.7 (38.3)	1618.7 (36.8)	0.571
	≥2	2383 (61.8)	325 (60.5)		2709.3 (61.7)	2783.7 (63.2)	
Aspirin usage		2000 (51.9)	263 (49.0)	0.229	2263.9 (51.5)	2260.2 (51.3)	0.946
Statin usage		1653 (42.9)	223 (41.5)	0.591	1877.8 (42.7)	1946.2 (44.2)	0.608
Smoking status							
	Never smoked	1684 (43.7)	229 (42.6)	0.906	1911.0 (43.5)	1811.7 (41.2)	0.648
	Former smoker	1637 (42.4)	232 (43.2)		1868.1 (42.5)	1982.8 (45.0)	
	Current smoker	536 (13.9)	76 (14.2)		613.9 (14.0)	607.9 (13.8)	
Baseline CKD		1045 (27.1)	223 (41.5)	<0.001	1265.5 (28.8)	1220.7 (27.7)	0.64
Previous CVD		778 (20.2)	113 (21.0)	0.679	890.6 (20.3)	888.6 (20.2)	0.969
Cancer history		465 (12.1)	92 (17.1)	0.001	555.4 (12.6)	547.3 (12.4)	0.897
eGFR, mL/min/1.73 $m^{2}$		72.43 (20.23)	66.15 (23.05)	<0.001	71.68 (20.62)	72.22 (21.07)	0.63
Serum creatinine, mg/dL		1.07 (0.33)	1.12 (0.46)	0.001	1.08 (0.35)	1.06 (0.34)	0.466
CHR, mg/dL		189.92 (41.14)	193.75 (44.99)	0.046	190.33 (41.14)	187.91 (44.21)	0.304
GLUR, mg/dL		99.18 (13.76)	97.01 (14.07)	0.001	98.94 (13.66)	99.08 (14.09)	0.867
HDL, mg/dL		52.46 (14.17)	55.78 (15.78)	<0.001	52.87 (14.33)	53.09 (15.24)	0.792
TRR, mg/dL		126.46 (89.35)	118.91 (68.05)	0.06	125.44 (86.85)	122.74 (80.84)	0.621
Urine albumin-to-creatinine ratio, mg/g		42.00 (177.37)	55.61 (184.60)	0.097	43.86 (180.54)	43.86 (156.92)	1

The data are presented as mean (standard deviation) or number (percentage). To convert values for creatinine to micromoles per liter, multiply by 88.4. To convert values for cholesterol to millimoles per liter, multiply by 0.02586. To convert values for triglycerides to millimoles per liter, multiply by 0.01129. To convert values for glucose to millimoles per liter, multiply by 0.05551. The body mass index (BMI) is calculated as weight in kilograms divided by the square of height in meters. BMI, body mass index; SBP, systolic blood pressure; DBP, diastolic blood pressure; FRS, Framingham risk score; CKD, chronic kidney disease; eGFR, estimated glomerular filtration rates; CVD, cardiovascular disease; CHR, Cholesterol; TRR, Triglycerides; GLUR, Glucose; HDL, high-density lipoprotein cholesterol direct; IPTW, inverse probability of treatment weighting.

### 3.2 Relationship between Anemia and Outcomes 

**Fig. 2.**
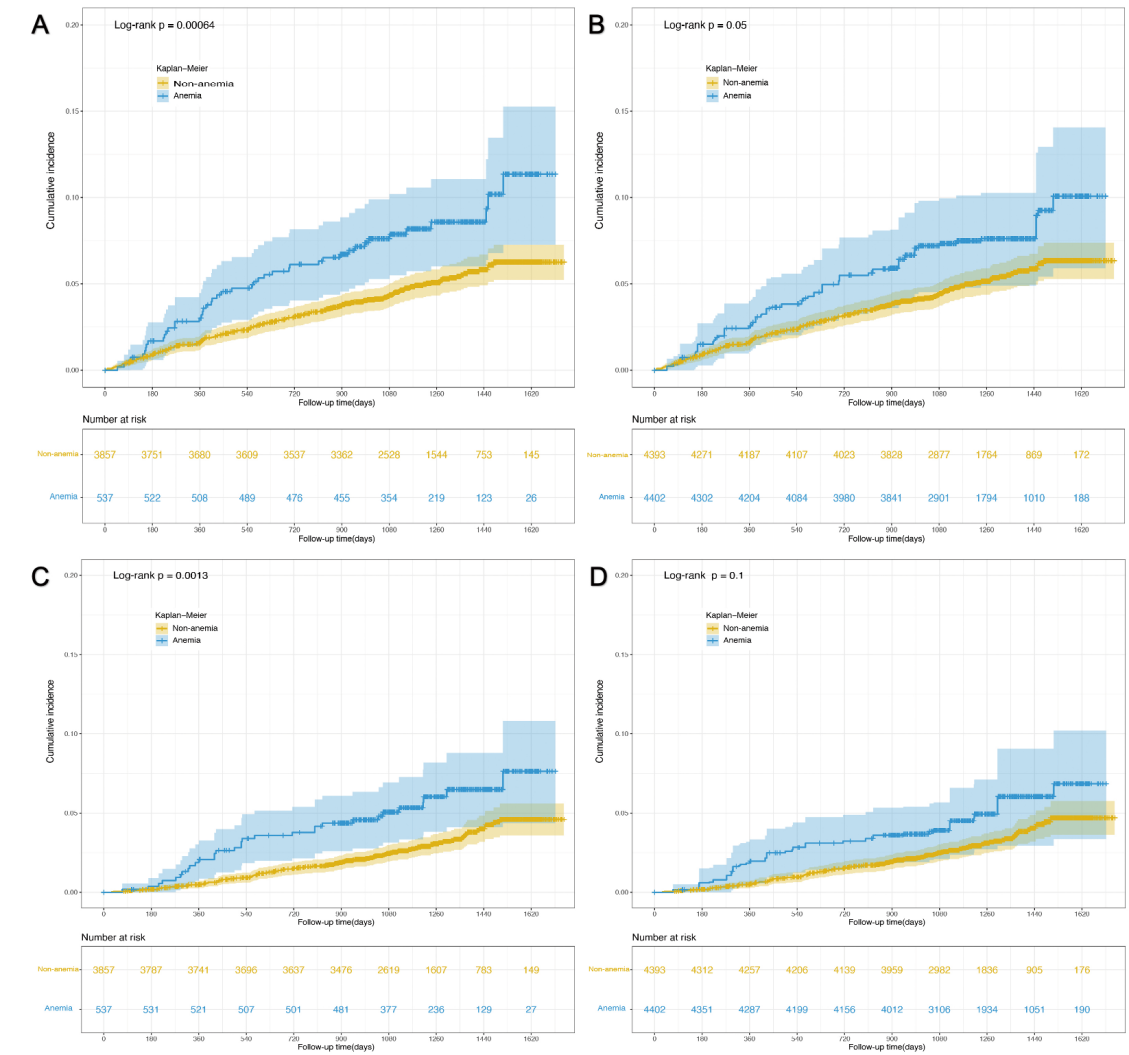
**Cumulative incidence plot of time to outcome events by anemia status**. (A) Impact of anemia on composite cardiovascular events in the pre-matched population. (B) Impact of anemia on composite cardiovascular events in post-matched population. (C) Impact of anemia on all-cause death in the pre-matched population. (D) Impact of anemia on all-cause death in the post-matched population.

Table 2 shows us the HRs for the association between anemia and outcomes. The presence of anemia was strongly associated with composite cardiovascular events after adjusting for potential confounders (HR 1.66, 95% CI 1.18–2.34, *p* = 0.004). The association remained statistically significant when we adjusted for propensity score (HR 1.61, 95% CI 1.14–2.27, *p* = 0.007) and did not change much even in the population after IPTW (HR 1.55, 95% CI 1.06–2.27, *p* = 0.024). The cumulative incidence plot indicates anemia was associated with the development of composite cardiovascular events, though the difference became marginally significant (*p* = 0.05) in the population after IPTW (Fig. 2A,B). As for the secondary outcome, participants with anemia had a higher rate of all-cause death compared with those without anemia (Table 2). The HR of all-cause death for participants with anemia compared with those without anemia was 1.75 (95% CI 1.15–2.66, *p* = 0.009) when adjusted for potential confounders, 1.67 (95% CI 1.09–2.54, *p* = 0.018) when adjusted for propensity score, and 1.61 (95% CI 1.00–2.57, *p* = 0.049) in the population after IPTW. The cumulative incidence plot indicates anemia was only associated with incident all-cause death in the pre-matched population (*p* = 0.001) but not in the population after IPTW (*p* = 0.1, Fig. 2C,D).

**Table 2.** **Impact of anemia on outcomes among participants who received intensive blood pressure control**.

Outcomes	Non-Anemia	Anemia	HR (95% CI)			
	N = 3857	N = 537	Model 1	Model 2	Model 3	Model 4
Primary outcome	Reference					
Composite cardiovascular events	186 (4.8%)	45 (8.4%)	1.75 (1.26, 2.42)	1.66 (1.18, 2.34)	1.61 (1.14, 2.27)	1.55 (1.06, 2.27)
Secondary outcome						
All-cause death	116 (3.0%)	31 (5.8%)	1.89 (1.27, 2.81)	1.75 (1.15, 2.66)	1.67 (1.09, 2.54)	1.61 (1.00, 2.57)

In model 1, no confounder was adjusted. In model 2, confounders with *p* value < 0.05 between the 2 groups were adjusted (age, gender, eGFR, SBP, DBP, FRS, baseline CKD, race, serum creatinine, cancer history, cholesterol, blood glucose, and high-density lipoprotein). In model 3, we adjusted for the predicted probability score calculated by the logistic regression model. In model 4, the HR was calculated within the population after the inverse probability of treatment weighting. HR, hazard ratio; CI, confidence interval; eGFR, estimated glomerular filtration rates; SBP, systolic blood pressure; DBP, diastolic blood pressure; FRS, Framingham risk score; CKD, chronic kidney disease.

**Fig. 3.**
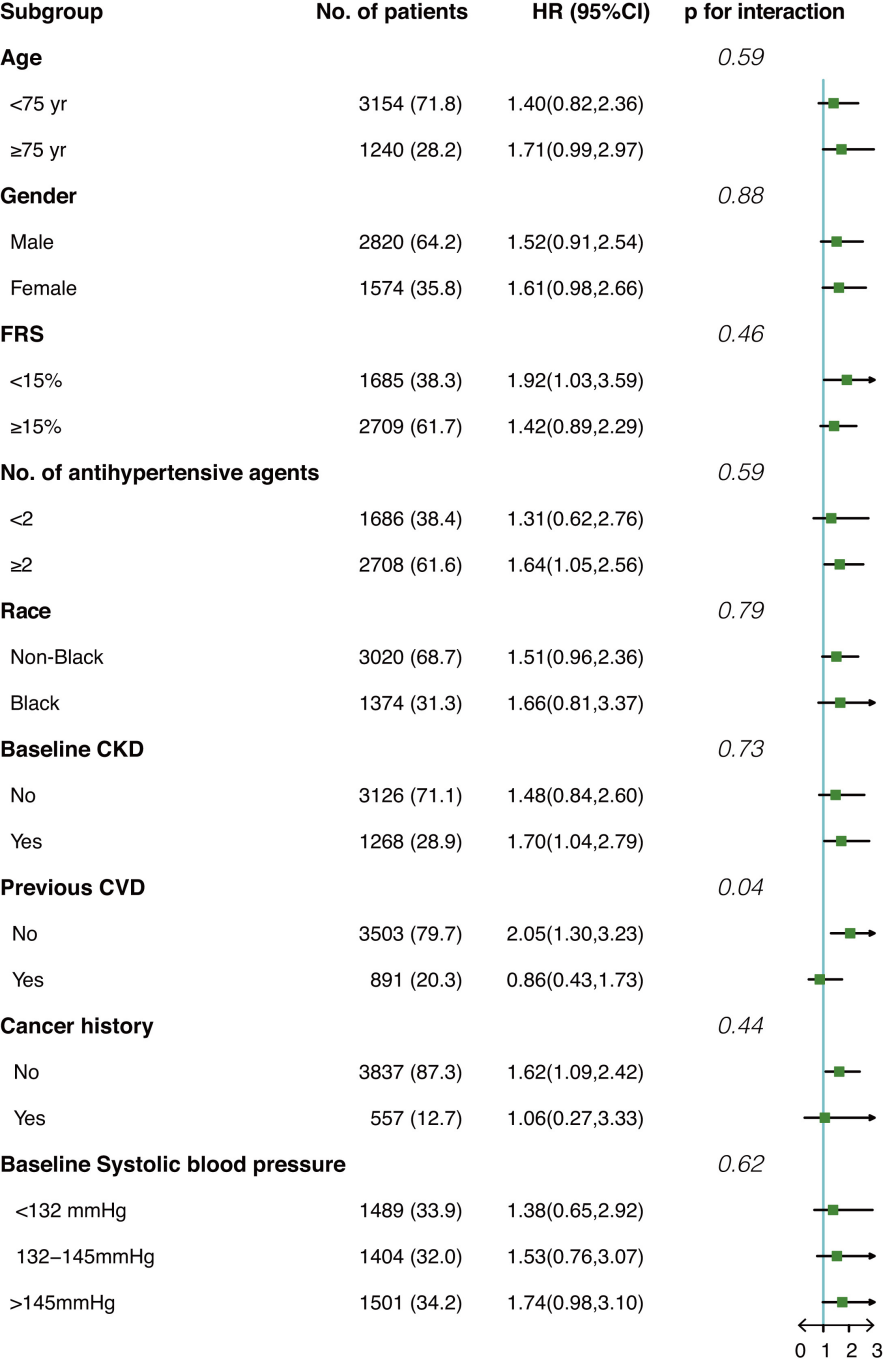
**Interaction effect between anemia and prespecified subgroups on composite cardiovascular events in the post-matched population**. HR, hazard ratio; FRS, Framingham risk score; CKD, chronic kidney disease; CVD, cardiovascular diseases.

Fig. 3 shows the interaction effect between anemia and prespecified groups on our primary outcome in the population after IPTW. Overall, no significant interaction effect was observed among all subgroups except for the subgroup of prior CVD (*p* for interaction = 0.04). Anemia seems to be a risk factor for composite cardiovascular events only among participants without prior CVD (HR 2.05, 95% CI 1.3–3.23) but not among participants with prior CVD (HR 0.86, 95% CI 0.43–1.73).

### 3.3 Sensitivity Analysis

First, anemia was not associated with composite cardiovascular events and all-cause death among participants treated with standard BP control (**Supplementary Table 1**). The HR of composite cardiovascular events and all-cause death for participants with anemia compared with those without anemia was 1.23 (95% CI 0.88–1.72, *p* = 0.221) and 1.19 (95% CI 0.78–1.82, *p* = 0.41) when adjusted for potential confounders, 1.15 (95% 0.83–1.62, *p* = 0.4) and 1.05 (95% CI 0.68–1.60, *p* = 0.84) when adjusted for propensity score, and 1.03 (95% 0.69–1.54, *p* = 0.89) and 1.08 (95% CI 0.66–1.78, *p* = 0.76) in the population after IPTW. Second, we divided the whole SPRINT participants into 2 groups (anemia and non-anemia) and investigated the interaction effect of anemia on the cardiovascular benefits of intensive BP control (**Supplementary Table 2**). The HR of composite cardiovascular events for participants who received intensive BP treatment compared with those who received standard BP treatment was 1.06 (95% CI 0.70–1.61, *p* = 0.77) for participants with anemia and 0.71 (95% CI 0.59–0.85, *p* < 0.001) for participants without anemia (interaction *p* = 0.08). The HR of all-cause death for participants who received intensive BP treatment compared with those who received standard BP treatment was 1.17 (95% 0.70–1.95, *p* = 0.56) for participants with anemia and 0.67 (95% CI 0.53,0.84, *p* < 0.001) for participants without anemia (interaction *p* = 0.05).

## 4. Discussion

Our study found that anemia was a significant risk factor for composite cardiovascular events and all-cause death among participants treated with intensive BP control in SPRINT. Anemia was associated with more than 50% higher risk of composite cardiovascular events and all-cause death, and this association was not found among participants treated with standard BP control in SPRINT. In the meantime, the cardiovascular protection effect of intensive BP control in SPRINT seems not to exist among participants with anemia, though the interaction *p* value did not reach <0.05. In light of these findings, our study indicated that intensive BP control of SBP <120 mmHg may not be suitable for participants with anemia. It’s better for physicians to acquire the medical history of anemia before considering implementing the intensive BP control strategy.

As compared with SPRINT, the Action to Control Cardiovascular Risk in Diabetes blood pressure trial (ACCORD BP) found intensive BP treatment was not able to reduce a composite of cardiovascular events, though they had the same SBP control target [18]. One major difference between the 2 trials is that ACCORD only enrolled participants with diabetes, whereas SPRINT excluded participants with diabetes. It remains unknown whether anemia can serve as a risk factor for composite cardiovascular events among participants treated with intensive BP control in ACCORD. It would be a good complement to our findings if there exists a positive link between anemia and risk for cardiovascular events among participants treated with intensive BP control in ACCORD.

There are some considerations that we have to explain the association between anemia and risk for composite cardiovascular events and all-cause death observed among participants treated with intensive BP control. First, chronic anemia has been reported to be associated with increased cardiac output, leading to ventricular dilation and left ventricular hypertrophy (LVH) [19, 20]. The structure change is known to be associated with an increased risk for cardiovascular events [21]. Although prior studies have indicated intensive BP control can improve cardiac structure, the improvement was not associated with reduced cardiovascular events [22, 23]. Moreover, the impact of intensive BP control on cardiac structure among participants with anemia and non-anemia was not known. Further investigation is needed in the future. Second, the blood capacity to carry oxygen is impaired among participants with low hemoglobin [24]. The blood supply to remote areas would be reduced when participants received intensive BP treatment; hence, the risk for cardiovascular events increased. Third, anemia may be the reflection of increased inflammatory status [25]. As we know, increased inflammatory status is associated with a higher risk for cardiovascular events. However, we were not able to adjust for biomarkers related to inflammatory status since they were not available in the SPRINT dataset. Fourth, previous studies suggested anemia can lead to decreased physical performance and cognition and increased frailty and dementia [26, 27, 28]. These factors may be the cause of increased cardiovascular risk and all-cause death. Fifth, studies indicated that the use of angiotensin-converting enzyme (ACE) inhibitors can depress the synthesis of erythropoietin [29], which can aggravate anemia. Among participants who received intensive BP treatment, this drug is commonly used. Sixth, the possibility that relative iron deficiency, rather than anemia per se, contributed to the development of cardiovascular disease cannot be ruled out.

The causes of anemia are diverse. Iron deficiency anemia has been reported to be the most common cause among the elderly population [30]. Besides, cancer of the large bowel, acute or chronic inflammatory diseases, and CKD are also associated with the development of anemia. Our subgroup analysis indicated anemia may not be the risk factor for composite cardiovascular events among participants with prior CVD (HR 0.86 95% CI 0.43–1.73, *p* for interaction 0.04). From our perspective, the prevalence of anemia may be higher among participants with prior CVD because of the acquired disability and impaired ability to absorb nutrition; therefore, the HR became non-significant when participants with anemia took a large proportion of the subgroup. After conducting an exploratory analysis, we found that the prevalence of anemia was higher among participants with prior CVD (13% versus 12%, *p* = 0.2), though it did not reach statistical significance. Because this study was a secondary analysis, we were not able to include unmeasured confounding factors, such as the treatment of anemia or CVD or lifestyle changes following CVD. These factors may also have played a role in our observation.

Several clinical studies have shown positive results with erythropoiesis-stimulating agents as a treatment of anemia to improve the outcomes of heart failure, end-stage kidney disease, and patients undergoing elective surgery [31, 32, 33]. As of now, whether the risk for cardiovascular events and all-cause death can be reduced with erythropoiesis-stimulating agents among patients treated with intensive BP control is not known. This question is important as more and more evidence indicates intensive BP control is feasible.

### Limitations

It is important to consider several limitations when interpreting the results of our study. First, this is a post-hoc analysis of SPRINT, and baseline characteristics between participants with anemia and non-anemia were unbalanced. Although we can balance baseline characteristics through IPTW, there may be other confounders that were not measured in SPRINT. Second, the anemia status was self-reported. The duration and degree of anemia can’t be adjusted in our model, so this may influence our observation. Prior treatment of anemia may also bias our observation. However, this kind of information was not provided in SPRINT. Third, as mentioned above, the causes of anemia are diverse. The association between anemia and outcomes may come from those undying diseases. Fourth, hemoglobin level was not measured in SPRINT. We were not able to analyze the impact of dynamic change in hemoglobin on our outcomes. Fifth, as shown in **Supplementary Fig. 1**, the BP of participants in the 2 treatment groups had a significant drop in the first 6 months after randomization. The dramatic change may increase the risk of cardiovascular disease, which was not considered in our study when investigating the impact of anemia. The landmark dynamic prediction model may help us gain insight into the dynamic changing effect of BP [34].

## 5. Conclusions

Anemia appears to be an independent risk factor for composite cardiovascular events and all-cause death among participants who received intensive BP control in SPRINT. Future studies are needed to investigate whether treatment of anemia with erythropoiesis-stimulating agents can improve CVD outcomes. In the meantime, the causes of anemia and their impact on CVD outcomes should also be investigated.

## Data Availability

All SPRINT anonymized data can be found at the National Heart, Lung and Blood Institute (NHLBI) Biologic Specimen and Data Repository (https://biolincc.nhlbi.nih.gov/home/).

## Supplementary Material

Supplementary material associated with this article can be found, in the online version, at https://doi.org/10.31083/j.rcm2501006.
