# Supplementary material for: Impact of Anemia on Cardiovascular Events and All-Cause Death Among Participants Who Received Intense Blood Pressure Treatment: A Secondary Analysis of SPRINT
Source: Rev Cardiovasc Med. 2024 Jan 8;25(1):6. doi: 10.31083/j.rcm2501006 (PMC11262380; doi:10.31083/j.rcm2501006)
Supplement: Supplementary file 1 [file 2153-8174-25-1-006-s1.docx]

**Online supplementary material**

**Supplementary Fig. 1. Dynamic change of blood pressure of participants with anemia and non-anemia after randomization.**


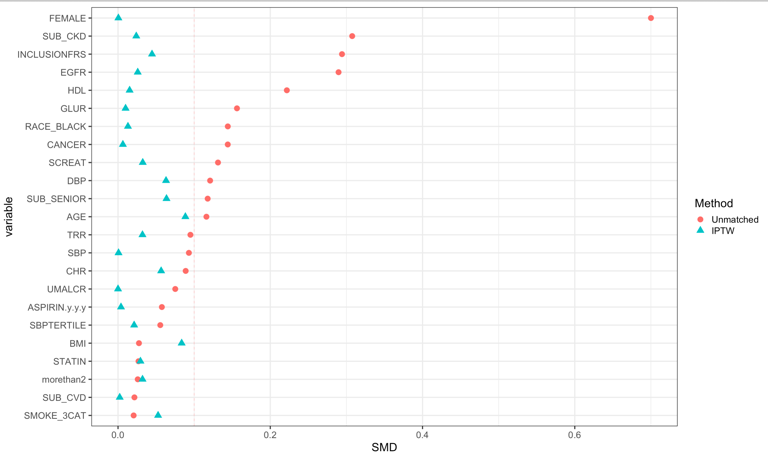


**Supplementary Fig. 2. Absolute standardized differences of baseline characteristics in pre- and post-matched population.**

Supplementary Table 1. Impact of anemia on outcomes among participants received standard blood pressure control.

|  |  |  | HR (95% CI) |  |  |  |
| --- | --- | --- | --- | --- | --- | --- |
| Outcomes | Non-Anemia | Anemia | Model 1 | Model 2 | Model 3 | Model 4 |
| Primary outcome | Reference |  |  |  |  |  |
| Composite cardiovascular events | 267 (7.0%) | 43 (7.9%) | 1.16(0.84,1.59) | 1.23(0.88,1.72) | 1.15(0.83,1.62) | 1.03(0.69,1.54) |
| Secondary outcome |  |  |  |  |  |  |
| All-cause death | 172 (4.5%) | 27 (5.0%) | 1.11(0.74,1.67) | 1.19(0.78,1.82) | 1.05(0.68,1.60) | 1.08(0.66,1.78) |

HR: hazard ratio; CI: confidence interval;

In model 1, no confounder was adjusted.

In model 2, confounders with P value <0.05 between the two groups were adjusted (age, gender, eGFR, SBP, DBP, FRS, baseline CKD, race, serum creatinine, cancer history, cholesterol, blood glucose, and high-density lipoprotein).

In model 3, we adjusted for the predicted probability score calculated by logistic regression model.

In model 4, the HR was calculated within the population after inverse probability of treatment weighting.

Supplementary Table 2. Interaction effect of anemia on cardiovascular benefits of intensive BP control among participants enrolled in SPRINT.

| Outcomes |  | Standard  BP treatment | Intensive  BP treatment | HR (95% CI) | P value | Interaction P |
| --- | --- | --- | --- | --- | --- | --- |
| Primary outcome |  | Reference |  |  |  |  |
| Composite cardiovascular events | Anemia | 43 (7.4%) | 46 (8.0%) | 1.06(0.70,1.61) | 0.77 | 0.08 |
|  | Non-anemia | 276 (6.7%) | 197 (4.8%) | 0.71(0.59,0.85) | <0.001 |  |
| Secondary outcome |  |  |  |  |  |  |
| All-cause death | Anemia | 27 (4.7%) | 32 (5.6%) | 1.17(0.70,1.95) | 0.56 |  |
|  | Non-anemia | 183 (4.5%) | 123 (3.0%) | 0.67(0.53,0.84) | <0.001 | 0.053 |

HR: hazard ratio; CI: confidence interval;
